# Retrospective Analysis of 50 Hemi-Keystone Flap Head and Neck Reconstructions with Scar Assessment

**DOI:** 10.3390/jcm15051888

**Published:** 2026-03-01

**Authors:** Wonseok Cho, Eun A Jang, Kyu Nam Kim

**Affiliations:** Department of Plastic and Reconstructive Surgery, Kangbuk Samsung Hospital, Sungkyunkwan University School of Medicine, Seoul 03181, Republic of Korea; wscws26@naver.com (W.C.); insane.blood.a@gmail.com (E.A.J.)

**Keywords:** keystone flap, local flap, skin defect, head defect coverage, neck defect coverage, reconstructive surgery

## Abstract

**Background/Objectives**: Skin and soft tissue defects of the head and neck are common challenges in plastic surgery and require reconstruction strategies tailored to defect size and depth. This study aimed to evaluate the clinical application and outcomes of the hemi-keystone flap (KF) technique and its modifications for head and neck reconstruction. **Methods**: A retrospective cohort study was conducted on 50 patients (36 males, 14 females; aged 9–92 years) who underwent hemi-KF reconstruction between September 2020 and March 2024. Data were collected on defect characteristics, flap design, surgical time, complications, scar outcomes, and follow-up duration. Scar outcomes were assessed using the Patient and Observer Scar Assessment Scale (POSAS). **Results**: The mean defect and flap sizes were 4.68 ± 4.14 cm^2^ and 11.79 ± 16.69 cm^2^, respectively. Single original hemi-KFs were used in 60% of cases, and double hemi-KFs in 32%. The mean flap-surgery duration was 29.04 ± 14.56 min. Partial wound dehiscence occurred in 6% of cases. The mean follow-up period was 6.34 ± 5.43 months. The mean POSAS scores were 15.30 ± 3.59 (patient) and 17.12 ± 3.70 (observer), indicating favorable scar outcomes and patient satisfaction. **Conclusions**: The hemi-KF technique and its modifications are reliable and versatile options for head and neck reconstruction, offering favorable functional and aesthetic outcomes.

## 1. Introduction

Skin and soft tissue defects in the head and neck are common challenges for plastic surgeons [1,2]. Various reconstructive options may be employed to cover these defects, and surgeons should select the most appropriate method in terms of both function and aesthetics on a case-by-case basis [3]. Generally, the size and depth of the defect are two crucial factors in determining which reconstructive option to choose. Small-sized defects can be covered by primary closure with undermining of the wound margin [4,5,6]. Any-sized defects with increasing depth may be covered by skin grafts [1]. Certain-sized defects with deeper depths and exposure of underlying structures should be managed by flap surgery rather than the skin grafting technique [1]. Free flaps are usually suitable for covering large and extensive defects, and local flaps are appropriate options to cover small- and moderate-sized defects in the human body [1,2].

Several local flaps have been developed to cover defects in various areas of the human body. Each local flap has its own design and movement for transfer to the defect, and most local flaps have limitations in either the size of the defect or the area to which they can be applied. Paradoxically, no unrivaled local flap technique can be applied for defects through whole-body areas. The keystone flap (KF), devised by Behan through his clinical experience of >300 successful cases in 2003, is a unique local flap that can be applied to various defects in nearly all parts of the human body, including the head, neck, trunk, and extremities [1,2,7,8,9]. Since Behan’s original four types of KF, several modifications, such as modified type II KF [10], omega variation closure (OVC) KF [11], Sydney Melanoma Unit modification (SMUM) KF [12], de-epithelialized KF [5], and hemi-KF [13], have been developed. These modifications could play an important role in broadening the scope of application of the KF technique and further enhancing its usefulness in the reconstructive surgical field.

Since 2019, the senior author (the corresponding author) of the present study has used the hemi-KF technique with modifications as a reconstructive modality. Herein, we present our experience with hemi-KF reconstruction for covering small-to-moderate-sized head and neck defects. The present report includes a detailed description of several useful hemi-KF modifications, accompanied by their illustrations and clinical photographs of representative cases. We additionally provide an analysis of the scar assessment results following hemi-KF reconstruction. This study aimed to facilitate the effective application of hemi-KFs with diverse defect coverage modifications to enhance the potential utility and feasibility of KF reconstruction in plastic surgery.

## 2. Materials and Methods

### 2.1. Ethical Statements

This study was approved by the Institutional Review Board of Kangbuk Samsung Hospital (approval number: 2024-07-022). The study protocol and research procedures adhered to the ethical standards outlined in the 1975 Declaration of Helsinki. Before the procedures and operations, all patients involved in the study provided written consent to publish their information and images in an online open-access format.

### 2.2. Study Design and Population

We enrolled patients who had received hemi-KF or its modifications for head and neck skin defects from September 2020 to March 2024, excluding patients treated with the original KF, other local flaps, or skin grafting. While ensuring patient anonymity, we reviewed electronic medical records and clinical photographs, using Microsoft Excel (in Microsoft 365) for data analysis. Data included information on defect details, flap characteristics, surgery time, survival, complications, scars, and follow-up.

### 2.3. Operative Techniques: Hemi-KF Reconstruction

**Figure 1:**
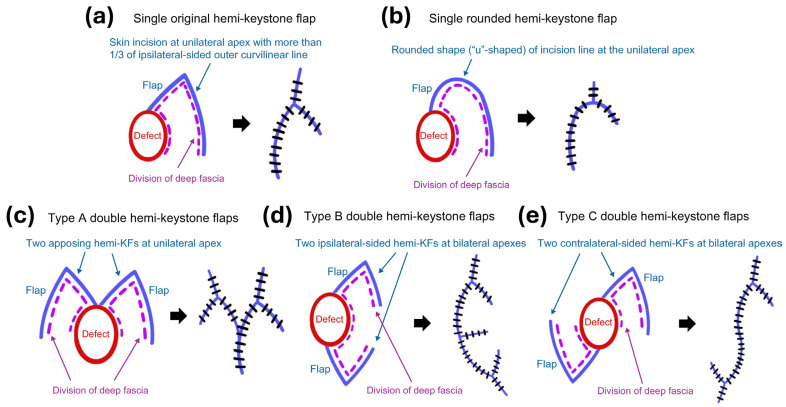
Schematic illustration of the hemi-keystone flap (hemi-KF) and its modifications: (**a**) Single original hemi-KF. (**b**) Single rounded hemi-KF. (**c**) Type A double hemi-KF. (**d**) Type B double hemi-KF. (**e**) Type C double hemi-KF.

In this study, five types of hemi-KFs were used: single original, single rounded, type A double, type B double, and type C double hemi-KFs. Figure 1 illustrates the five subtypes of hemi-KFs. The single original hemi-KF procedure involves a skin incision and division of the deep fascia at the unilateral apex, extending over more than one-third of the outer curvilinear line on the same side [13]. For the single rounded hemi-KF, the shape of the incision line at the unilateral apex is round (“u”-shaped) rather than angled (“v”-shaped), providing more rotational flap movement with the convenience of donor site closure. We classified three subtypes of double hemi-KFs as follows: type A double hemi-KFs involving two opposing hemi-KFs at the unilateral apex, type B double hemi-KFs consisting of two ipsilateral-sided hemi-KFs at bilateral apexes, and type C double hemi-KFs consisting of two contralateral-sided hemi-KFs at bilateral apexes. When performing a double hemi-KF technique, each case could involve the use of either an original hemi-KF or a rounded hemi-KF. Thus, both double original and double rounded hemi-KFs are feasible. At the time of flap design, three factors, including flap size, laxity of surrounding tissue (LST), and relaxed skin tension lines (RSTLs), should be considered [1,2]. Each flap was designed with its long axis parallel to the RSTLs and its width larger than that of the defect, whichever had more LST [1,2,9]. All types of hemi-KFs involve deep fascia division along the skin incision and minimal flap undermining to allow the flap to reach the defect without tension. We decided which type of hemi-KF should be administered intraoperatively rather than preoperatively. On a case-by-case basis, double hemi-KFs were designed, and the procedures were performed from the outset; if defect coverage was inadequate with a single hemi-KF, an additional hemi-KF was subsequently used. For each case, we first chose whether to use the original or rounded hemi-KF based on flap movement toward the defect. Next, for cases in which one flap was insufficient to cover the defect, we applied another hemi-KF in the area with adequate LST, which eventually formed a double hemi-KF, including types A, B, and C double hemi-KFs. Meanwhile, flap and defect areas were calculated as length × width (cm^2^) based on intraoperative measurements in all cases included in this study.

### 2.4. Evaluation of Final Scar Outcomes

We evaluated scar outcomes using the Patient and Observer Scar Assessment Scale (POSAS) at the last follow-up [14,15]. The senior author assessed the Observer Scar Assessment Scale (OSAS), and patients rated their scars using the Patient Scar Assessment Scale (PSAS). Both scales use a 10-point system to score six aspects: OSAS includes vascularity, pigmentation, thickness, pliability, relief, and surface area, whereas PSAS covers pain, itching, color, stiffness, thickness, and irregularity [14,15]. Scores range from 6 (normal skin) to 60 (worst scar). Objective scar ratings and overall patient satisfaction were also rated on a 10-point visual analog scale [14,15].

### 2.5. Statistical Analysis

#### 2.5.1. Software and Descriptive Statistics

All statistical analyses were performed using R software (version 4.0.5; R Foundation for Statistical Computing, Vienna, Austria). Continuous variables are presented as means ± standard deviations (SDs), and categorical variables are expressed as numbers and percentages (*n* (%)). Normality of continuous variables was assessed using the Shapiro–Wilk test.

#### 2.5.2. Comparative Analyses

For comparisons between independent groups, normally distributed continuous variables (e.g., PSAS and OSAS total scores) were analyzed using the independent Student’s *t*-test. Non-normally distributed continuous variables were analyzed using the Wilcoxon rank-sum test (Mann–Whitney U test). Overall patient satisfaction and objective scar rating were recorded on a 10-point scale and treated as ordinal variables; therefore, between-group comparisons were performed using the Wilcoxon rank-sum test. Categorical variables were compared using the chi-square test or Fisher’s exact test, as appropriate. A two-tailed *p*-value < 0.05 was considered statistically significant.

## 3. Results

Table 1 and Table 2 summarize the patient demographics and outcomes of this study. Overall, 50 (36 male and 14 female) patients aged 9–92 years (mean age ± SD, 49.16 ± 22.02 years) were selected. In terms of underlying diseases, 10 (20%) patients had diabetes mellitus, 21 (42%) had hypertension, 13 (26%) used aspirin, and 15 (30%) had a history of smoking. A total of 50 hemi-KF procedures were performed by a single surgeon, the senior author of the present study. The causes of defects were trauma in 33 patients (66%), skin tumors in 10 (20%), postoperative wound dehiscence in 3 (6%), skin infection in 2 (4%), and pressure ulcers in 2 patients (4%). The defect locations were the scalp and forehead in 16 patients (32%), nose in 11 (22%), cheek in 5 (10%), eyelid area in 5 (10%), lip area in 4 (8%), auricular area in 7 (14%), and neck area in 2 patients (4%). The mean defect size was 4.68 ± 4.14 cm^2^, ranging from 0.5 to 14 cm^2^. The smallest defect was 0.5 × 1 cm^2^ in the postauricular area, and the largest defect was 3.5 × 4 cm^2^ on the scalp. The mean flap size was 11.79 ± 16.69 cm^2^, ranging from 0.5 to 40.5 cm^2^. The smallest flap was a 0.5 cm^2^ sized type C double hemi-KF in the philtrum, and the largest flap was a 40.5 cm^2^ sized type A double hemi-KF on the scalp. Regarding types of hemi-KFs, single original hemi-KFs were used in 30 cases (60%), and single rounded hemi-KFs were used in 4 cases (8%). Double hemi-KFs were used in 16 cases (32%), including 2 cases (4%) of type A, 11 (22%) of type B, and 3 (6%) of type C. The mean flap-surgery duration, defined as the time elapsed from the initiation of flap elevation to the completion of flap inset and donor site closure, was 29.04 ± 14.56 min, ranging from 15 to 68 min. Postoperative complications included partial wound dehiscence owing to marginal maceration in 3 patients (6%), which were treated by conservative wound care without further surgical intervention in 2 patients and by surgical wound repair with additional hemi-KF coverage in 1 patient. In this study, patients experienced no major complications, such as bleeding, hematoma formation, partial or complete flap loss, or surgical site infection. The mean final follow-up period was 6.34 ± 5.43 months, ranging from 2 to 28 months.

**Figure 2:**
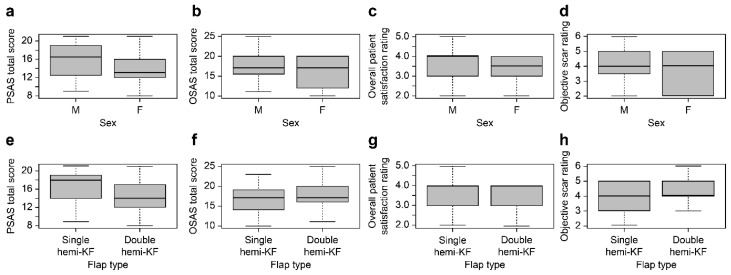
Comparison graphs of the PSAS total scores, OSAS total scores, overall patient satisfaction, and objective scar rating between (**a**–**d**) male and female patients, as well as (**e**–**h**) patients who underwent single hemi-KF and double hemi-KF reconstruction in the present study.

The mean PSAS and OSAS total scores of all patients were 15.30 ± 3.59 and 17.12 ± 3.70, respectively. The mean objective scar rating and overall satisfaction of all patients were 3.38 ± 0.87 and 3.98 ± 1.02, respectively. The mean values of the PSAS total scores, OSAS total scores, overall patient satisfaction, and objective scar rating according to sex were 15.36 ± 3.60 and 15.08 ± 3.60, 17.22 ± 3.67 and 17.28 ± 3.79, 3.40 ± 0.86 and 3.34 ± 0.89, and 4.020 ± 0.989 and 4.02 ± 0.10 for male and female patients, respectively, with no significant differences observed (Table 3). The mean values of the PSAS total scores, OSAS total scores, overall patient satisfaction, and objective scar rating according to the hemi-KF type were 15.22 ± 3.60 and 15.38 ± 3.67, 3.37 ± 0.89 and 3.42 ± 0.87, 17.16 ± 3.77 and 17.10 ± 3.64, and 3.97 ± 1.04 and 4.00 ± 1.00 for patients who underwent single and double hemi-KF procedures, respectively, with no significant differences observed (Table 4). Figure 2 shows the comparison graphs of the PSAS total scores, OSAS total scores, overall patient satisfaction, and objective scar ratings according to sex (male vs. female) and flap type (single hemi-KF vs. double hemi-KFs). The following Section 3.1, comprising case presentations, delineates representative instances that exemplify the outcomes of hemi-KF, along with its modified techniques delineated by specific head and neck areas, as investigated in this study.

### 3.1. Case Presentations of Head and Neck Defect Coverage with the Hemi-KF with Its Modifications

#### 3.1.1. Forehead Defect Coverage with a Single Original Hemi-KF

**Figure 3:**
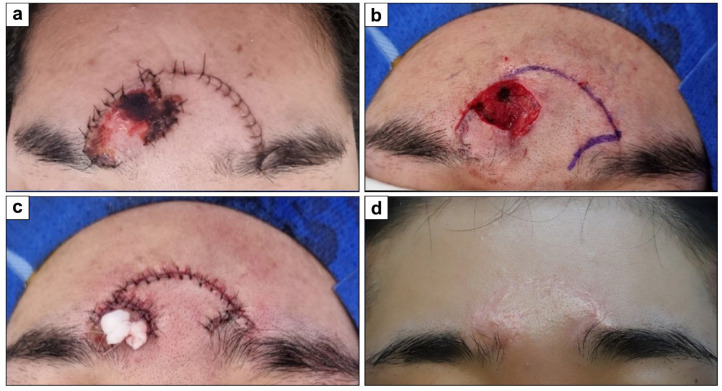
Clinical photographs of forehead defect coverage with a single original hemi-keystone flap (hemi-KF): (**a**) Forehead skin necrosis. (**b**) Post-debridement final defect and design of a single original hemi-KF. (**c**) Successful flap coverage. (**d**) Twelve-month follow-up photograph.

A 22-year-old woman with no prior health issues had forehead skin necrosis after a skin avulsion injury. A 2.5 × 2.5 cm^2^ defect was covered with a 3 × 5 cm^2^ hemi-KF along previous suture lines. The flap survived without complications. At the 12-month final follow-up, the PSAS and OSAS scores were 12 and 13, respectively, with overall patient satisfaction and objective scar ratings of 4 each. Figure 3 shows clinical photographs of this case.

#### 3.1.2. Parietal Scalp Defect Coverage with a Single Rounded Hemi-KF

**Figure 4:**
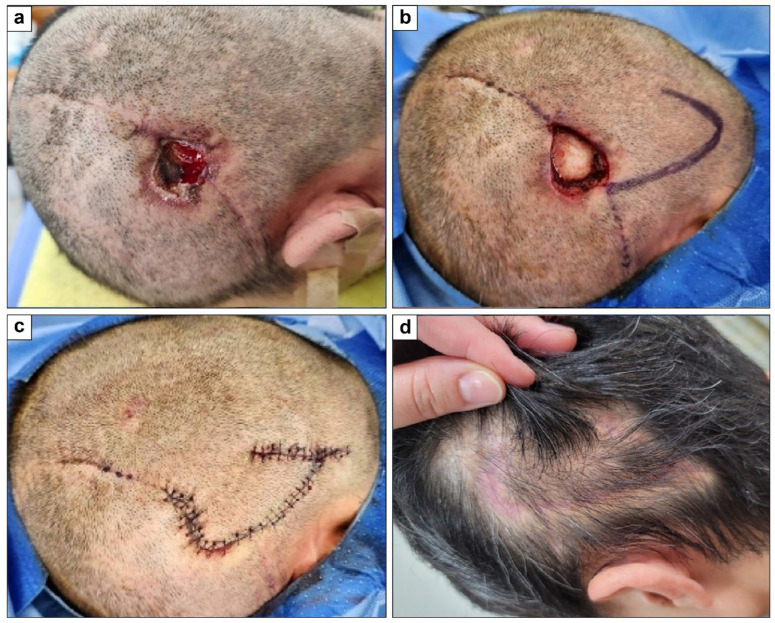
Clinical photographs of parietal scalp defect coverage with a single rounded hemi-keystone flap (hemi-KF): (**a**) Full-thickness skin defect. (**b**) Post-debridement final defect and design of a single rounded hemi-KF. (**c**) Successful flap coverage. (**d**) Six-month follow-up photograph.

A 39-year-old man with no health issues had a 3 × 3.5 cm^2^ scalp defect from wound dehiscence. A 5 × 6 cm^2^ hemi-KF from the occipital area was used to cover the defect, with direct closure of the donor site. The flap survived without complications. At the 6-month final follow-up, the PSAS and OSAS scores were 17 and 22, respectively, with overall patient satisfaction and objective scar ratings of 3 and 5, respectively. Figure 4 shows clinical photographs of this case.

#### 3.1.3. Lateral Cheek Defect Coverage with a Single Rounded Hemi-KF

**Figure 5:**
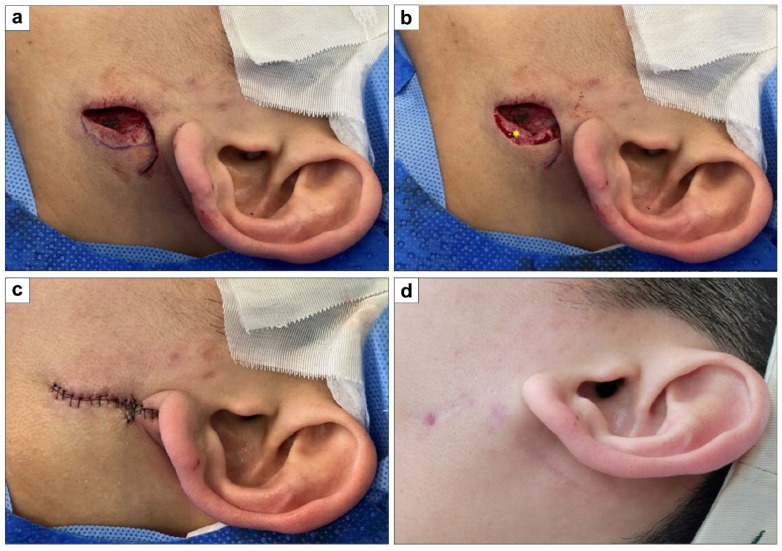
Clinical photographs of lateral cheek defect coverage with a single rounded hemi-keystone flap (hemi-KF): (**a**) Final defect and design of a single rounded hemi-KF. (**b**) Partial skin flap de-epithelialization (yellow star). (**c**) Successful flap coverage. (**d**) Fifteen-month follow-up photograph.

A 26-year-old man with no health issues had a 1.5 × 2 cm^2^ defect after excising an infected epidermoid cyst on his cheek. The defect was covered using a 1.5 × 1.5 cm^2^ hemi-KF from the posterior side, with direct closure of the donor site. The flap survived without complications. At the 15-month final follow-up, the PSAS and OSAS scores were 16 and 20, respectively, with overall patient satisfaction and objective scar ratings of 4 each. Figure 5 shows clinical photographs of this case.

#### 3.1.4. Forehead Defect Coverage with Type A Double Original Hemi-KFs

**Figure 6:**
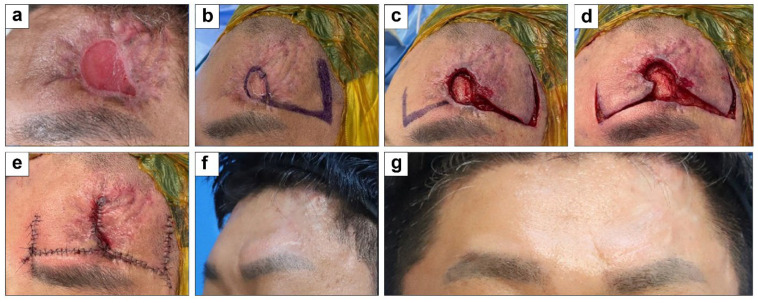
Clinical photographs of forehead defect coverage with type A double original hemi-keystone flaps (hemi-KFs): (**a**) Forehead defect. (**b**) Design of a single original hemi-KF. (**c**) Design of an additional original hemi-KF. (**d**,**e**) Successful flap coverage. (**f**,**g**) A 28-month follow-up photograph.

A 37-year-old man with no health issues had a 2 × 3 cm^2^ forehead defect from trauma, with the surrounding tissue having reduced elasticity owing to scarring. Initially, we used a 5 × 6 cm^2^ hemi-KF on the lateral side, resulting in insufficient defect coverage. Thus, we subsequently used a 4 × 5 cm^2^ hemi-KF on the medial side, which finally showed type A double original hemi-KFs. Surgery lasted 47 min, and all flaps survived without complications. At the 28-month follow-up, the PSAS and OSAS scores were 18 and 20, respectively, with overall patient satisfaction and objective scar ratings of 4 and 5, respectively. Figure 6 shows clinical photographs of this case.

#### 3.1.5. Posterior Ear Defect Coverage with Type B Double Rounded Hemi-KFs

**Figure 7:**
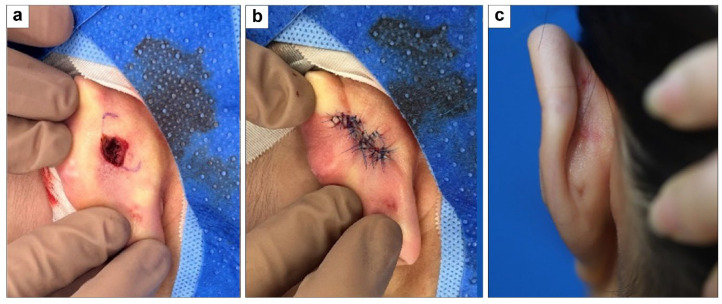
Clinical photographs of posterior ear defect coverage with type B double-rounded hemi-keystone flaps (hemi-KFs): (**a**) Final defect and design of type B rounded hemi-KFs. (**b**) Successful flap coverage. (**c**) Five-month follow-up photograph.

A 19-year-old woman with no health issues had a 1 × 1 cm^2^ ear defect from a skin infection. The defect was covered with two 0.5 × 1 cm^2^ type B double-rounded hemi-KFs from the upper and lower sides, with direct closure of the donor site. All flaps survived without complications. At the 5-month final follow-up, the PSAS and OSAS scores were 13 and 11, respectively, with overall patient satisfaction and objective scar ratings of 2 each. Figure 7 shows clinical photographs of this case.

#### 3.1.6. Philtrum Defect Coverage with Type C Double Rounded Hemi-KFs

**Figure 8:**
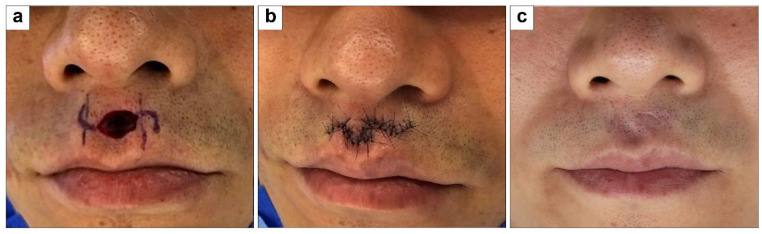
Clinical photographs of philtrum defect coverage with type C double rounded hemi-keystone flaps (hemi-KFs): (**a**) Final defect and design of type C rounded hemi-KFs. (**b**) Successful flap coverage. (**c**) Three-month follow-up photograph.

A 43-year-old man with no health issues had a 1 × 1 cm^2^ philtrum defect from trauma. The defect was covered with two 0.5 × 0.5 cm^2^ type C double-rounded hemi-KFs from the lateral sides, with direct closure of the donor site. All flaps survived without complications. At the 3-month follow-up, the PSAS and OSAS scores were 18 and 21, respectively, with overall patient satisfaction and objective scar ratings of 4 and 5, respectively. Figure 8 shows clinical photographs of this case.

#### 3.1.7. Posterior Ear Defect Coverage with a Single Original Hemi-KF Followed by an Additional Single Original Hemi-KF

**Figure 9:**
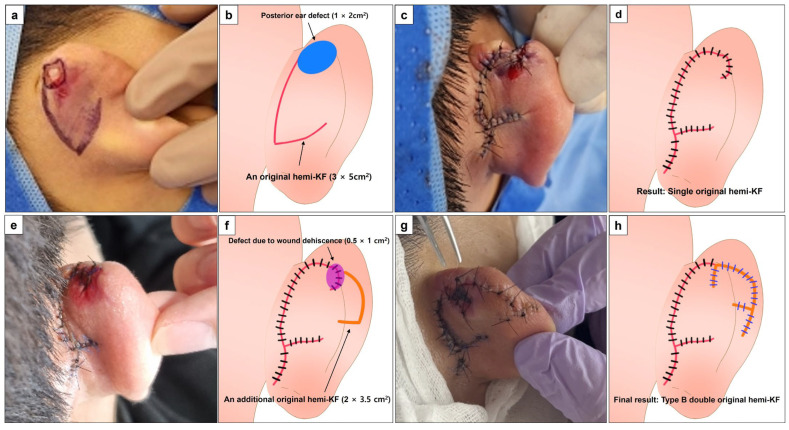
Clinical photographs and schematic illustrations of posterior ear defect coverage with a single original hemi-keystone flap (hemi-KF) followed by an additional single original hemi-KF: (**a**–**d**) Procedures for the first single hemi-KF. (**e**–**h**) Procedures for the second single hemi-KF.

**Figure 10:**
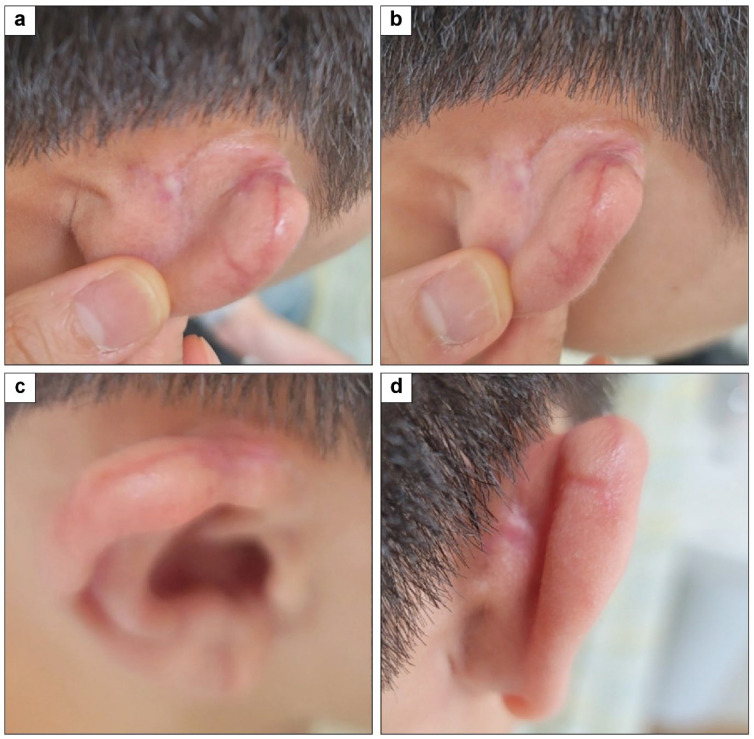
Four-month follow-up photographs of posterior ear defect coverage with a single original hemi-keystone flap (hemi-KF) followed by an additional single original hemi-KF: (**a**,**b**) upper oblique view, (**c**) upper view, and (**d**) posterior view of the right ear.

A 23-year-old heavy smoker had a 1 × 2 cm^2^ full-thickness skin defect on his posterior ear from trauma. Initially, a 3 × 5 cm^2^ hemi-KF covered the defect; however, dehiscence occurred 5 days later, creating a 0.5 × −1 cm^2^ defect. A second 2 × 3.5 cm^2^ hemi-KF was used to cover this new defect, resulting in the final shape of the type B double hemi-KFs. The second flap healed without complications. At the 4-month final follow-up, the PSAS and OSAS scores were 14 and 17, respectively, with overall patient satisfaction and objective scar ratings at 3 and 4, respectively. Figure 9 and Figure 10 show clinical photographs of this case.

## 4. Discussion

Tissue loss is a common challenge in reconstructive surgery, where optimal outcomes are achieved by using tissue similar to the original tissue [1,2,7,8]. Local flap techniques are ideal for covering defects with tissue that closely matches the surrounding area, leading to better aesthetic and functional results [1,9]. Although free flap techniques are essential for extensive defects or specialized tissue reconstruction, local flaps generally provide superior results for small-sized to moderate-sized defects, especially in the head and neck, where aesthetic considerations are crucial [1]. Therefore, this study assessed the effectiveness of the hemi-KF technique in treating small to moderate-sized skin defects in the head and neck in 50 cases.

The KF, with its various modifications, holds significant importance in the field of reconstructive surgery, particularly because of its intuitive flap design, ease of acquisition with a minimal learning curve, excellent reproducibility, and stable flap hemodynamics, which have established the KF as a popular reconstructive modality for covering various defects [1,5,7,8,9,10,11,12]. The hemi-KF, initially conceived by Petukhova et al. in 2020, is formally described as the V-Y hemi-keystone advancement flap [13]. Petukhova et al. elucidated that this hemi-KF entails unilateral incisions along the curvilinear aspect of the flap, as necessary, to achieve closure of the defect through minimal tissue incision, compared with that of the original keystone flap [13]. They stated that through such minimal tissue incision, the hemi-KF can preserve the aforementioned advantages of Behan’s original KF while reducing donor site morbidity, compared with that of the original KF, and enhancing the efficiency of flap surgery [13].

Meanwhile, in 2022, Hifny and Park presented a case series of modified rotation hemi-KFs emphasizing rotational flap movement [16,17]. They stated that the original KF may be vulnerable to problematic wound healing and tissue necrosis owing to the relatively considerable incision area required compared to the defect size, as well as the maximal tension experienced by the central portion of the flap during closure of the defect [16,17]. Although some modifications, such as the SMUM KF and OVC KF, have been devised to address these vulnerabilities of the original KF [16,17], they still entail technically substantial incision requirements and relatively time-consuming procedures [16,17]. Hifny and Park also suggested that the rotation hemi-KF technique can enhance flap mobility by extending the range of flap undermining and adding further rotational movement, thereby achieving defect closure with reduced tension [16,17]. The difference between the rotation hemi-KF demonstrated by Hifny and Park and the hemi-KF demonstrated by Petukhova et al. lies in the emphasis on the importance of flap rotation movement, which is determined by the extent of flap undermining. However, both methods fundamentally represent the same technique. The hemi-KF can be clearly characterized as having a simpler and more intuitive design than the original KF while ensuring stable blood flow in the flap and allowing for increased mobility of the flap. Accordingly, the hemi-KF offers the advantages of minimizing incisions, reducing the risk of complications, and decreasing the duration of flap surgery [13,16].

In this study, we aimed to secure systematic diversity in the application of hemi-KF reconstruction by employing the original hemi-KF and its four modifications, including the rounded, type A double, type B double, and type C double hemi-KF, to cover small to moderate-sized defects in the head and neck. Although some may argue that the original hemi-KF design resembles the traditional Limberg or rhomboid flap in terms of shape and configuration [18,19,20], the original hemi-KF possesses distinct characteristics that differentiate it from these flaps [16]. The original hemi-KF is designed parallel to the long axis of the defect’s edge, whereas the Limberg flap is designed perpendicular to the long axis at the short axis of the defect [19,20]. The former primarily advances forward with additional rotation, while the latter moves transposed and rotated [16,17,18,19]. Furthermore, the former requires minimal flap undermining for the flap movement to cover the defect, whereas the latter requires substantial flap undermining corresponding to the size of the designed flap [16]. Based on these differences, the hemi-KF is believed to exhibit more robust flap perfusion and a more stable wound-healing process than other random pattern flaps because it provides better tissue mobility by adding a greater rotation movement to close the wound defect with minimal tension [16]. The rounded hemi-KF is characterized by the modification of the angular design of the apex region of the original hemi-KF into a rounded design, thereby imparting greater rotational movement to the flap and facilitating ease of suturing at the donor site. Some may argue that this rounded hemi-KF is similar to conventional rotational flaps. However, the rounded hemi-KF follows the fundamental design aspects of the hemi-KF, exhibiting not only rotational flap movement but also advancement movement, thus distinguishing itself from a simple rotational flap. Aside from the rounded design at the flap apex, the surgical technique for the rounded hemi-KF is essentially identical to that for the original hemi-KF. In this study, we performed a total of 42 original hemi-KF procedures and 8 rounded hemi-KF procedures, without distinguishing between single and double hemi-KF, and achieved satisfactory outcomes without any significant complications in all cases. We believe that no absolute criterion exists for choosing the original or rounded hemi-KF for defect coverage. The decision should be made on a case-by-case basis, considering the required flap movement for defect coverage, the minimization of wound tension during flap suturing, and the final shape and direction of the suture lines after defect coverage, which influence uneventful wound healing and the formation of aesthetically pleasing scars. In certain cases, two hemi-KFs may be more advantageous than a single hemi-KF to cover defects. Thus, in cases of relatively large defects or defects with insufficient surrounding tissue, the application of two hemi-KFs is superior to that of a single large hemi-KF. This approach minimizes tension during wound closure and promotes uneventful wound healing.

As previously mentioned, we categorized the use of double hemi-KFs into three types based on the position of the additional hemi-KF. Key factors to consider when designing any local flap include the laxity and availability of the surrounding tissue, relaxed skin tension lines, and major anatomical landmarks [1,2,9]. The flap should be designed in an area around the defect with the greatest tissue laxity and availability, aligned as parallel as possible to the relaxed skin tension lines while preserving major anatomical landmarks [1,9]. Two application methods should be considered when using the double hemi-KF. One method involves initially designing two hemi-KFs, considering the key factors mentioned above, and completing the flap procedure without adding any other flaps midway through the final defect. The other method involves initially performing a single hemi-KF for the final defect; if coverage with one hemi-KF is insufficient, an additional hemi-KF is subsequently performed. In this study, the choice between these two methods was determined on a case-by-case basis, based on the surgeon’s preference. As depicted in Figure 7, the former method was applied to a final defect measuring 1 × 1 cm^2^ in the posterior ear region. From the outset, two rounded hemi-KFs measuring 0.5 × 1 cm^2^ were designed, and flap surgery proceeded without the need for additional flap design midway. In the case depicted in Figure 6, the latter method was applied to a final defect measuring 2 × 3 cm^2^ in the forehead region. Initially, a hemi-KF measuring 5 × 6 cm^2^ was utilized, and a second hemi-KF measuring 4 × 5 cm^2^ was added to cover the remaining defect that could not be completely covered by the first flap, thus completing the flap surgery.

In this study, we utilized 16 double hemi-KFs and achieved satisfactory outcomes without any notable complications. The type A double hemi-KF, in terms of its design, can be regarded as utilizing only half of the flap from Behan’s original type III KF (double opposing KFs). Because type A double hemi-KFs employ two hemi-KFs on the same side of the edge of the defect, they require a donor site with more surplus tissue than other subtypes of double hemi-KFs. In this study, we used type A double hemi-KFs to transfer a flap from the forehead, which has greater tissue laxity and elasticity than the scalp, to the defect area in the frontal and anterior scalp regions caused by trauma. When tissue redundancy surrounding the defect is asymmetrically distributed, and two intermediate-sized flaps are deemed more effective than a single large flap, type A double hemi-KFs can be a suitable reconstructive option. Regarding type B double hemi-KFs, some may argue that the flap design and shape are identical to the SMUM KF devised by Moncrieff et al. [12], which is a variant of the original KF and involves the maintenance of a skin bridge along the outer curvilinear line. However, type B double hemi-KFs are uniquely advantageous, as each hemi-KF is designed with different shapes and sizes tailored to the defect, considering the characteristics of the surrounding tissue. This personalized approach allows for more customized treatment than that with the SMUM KF. Type C double hemi-KFs involve the use of two hemi-KFs positioned at each edge of the defect, facing opposite sides. This design is similar to the O-to-Z rotation flap, a well-established technique among rotation flap procedures [21,22,23,24]. However, similar to other double hemi-KF subtypes, type C double hemi-KFs involve both rotation and advancement of the flap, ensuring greater stability in flap perfusion by minimizing flap undermining.

One of the concerns of surgeons when reconstructing skin and soft tissue defects using the local flap technique is the postoperative scar shape, including scar size and length. The hemi-KF technique utilizes half of the original KF design, resulting in a skin incision that is also half the length of the original KF procedure [13,16]. Consequently, this technique inherently offers the advantage of smaller scar sizes and lengths postoperatively. Nevertheless, compared with primary closure, the use of a flap inevitably requires additional incisions at the donor site. Therefore, surgeons must meticulously design flaps based on the final appearance of the scar. Additionally, for cases where double hemi-KFs were performed, the resulting scars were similar to those of the original KFs. Therefore, as in other local flap procedures, a careful approach must be adopted to avoid scarring. In this study, we designed a flap parallel to the RSTL to minimize postoperative scar formation. Additionally, we recommended basic postoperative scar management for the patients in this study. After suture removal, patients were instructed to undergo taping therapy for 2 weeks and to subsequently use scar ointment and silicone scar sheets for 3 months. Few studies have evaluated scarring after KF procedures. However, one previous study reported the POSAS scores of 20 patients who underwent KF reconstruction, with the mean PSAS and OSAS total scores being 11.0 ± 10.0 and 10.9 ± 2.4, respectively. In our cases, the POSAS results for the final scar assessment showed mean PSAS and OSAS total scores of 15.3 ± 3.59 and 17.12 ± 3.70, respectively. The overall patient-reported scar outcome (mean overall patient satisfaction, 3.38 ± 0.87) and third-party evaluation of the scar (mean objective scar rating, 3.98 ± 1.02) also showed favorable results.

In this study, no significant differences were observed in the mean values of the PSAS total score (*p* = 0.21), OSAS total score (*p* = 0.33), overall patient satisfaction (*p* = 0.08), or objective scar rating (*p* = 0.55) between patients who received single hemi-KFs and those who received double hemi-KFs. These results suggest that the increased extent of scarring attributed to the additional incision line required in flap surgery does not necessarily have a negative impact on the scar assessment outcomes of head and neck reconstructions using the hemi-KF technique. In this study, no significant sex differences were observed in the mean values of the PSAS total score (*p* = 0.08), OSAS total score (*p* = 0.13), overall patient satisfaction (*p* = 0.74), or objective scar rating (*p* = 0.08). These results also suggest that sex is not a significant factor influencing scar assessment outcomes following the reconstruction of head and neck defects using the hemi-KF technique. To the best of our knowledge, no study has compared scar assessments following KF procedures based on patient sex and flap type. Our results may serve as a reference for surgeons considering KFs in the reconstruction of head and neck defects, particularly for postoperative scarring.

Despite the successful reconstruction of various head and neck defects using the hemi-KF technique and its modifications in this study, some limitations must be considered. This was a retrospective case series, which inherently carries a lower level of evidence owing to the lack of a control group. This study had a relatively small sample size and involved heterogeneous cases. Consequently, inadvertent selection bias and confounding bias may have been introduced. Furthermore, this study was conducted at a single center, and the follow-up periods varied and were relatively short in some cases, potentially limiting the generalizability of the study findings. In addition, all procedures were performed by a single surgeon, and the study reflects the experience of one operator. Although this ensures technical consistency, it may introduce operator-dependent bias and may limit the external validity and reproducibility of the findings in different clinical settings. Moreover, outcome assessment and data interpretation may be influenced by single-investigator evaluation, and the absence of independent or multiple evaluators may represent an additional source of potential bias.

In particular, the follow-up intervals ranged broadly across patients, and scar assessments were performed at different postoperative time points. Because scar maturation evolves over time, outcomes evaluated at an early postoperative stage (e.g., 2–3 months) may not be directly comparable with those assessed at later stages (e.g., beyond 12 months). The absence of time-stratified subgroup analysis may therefore limit the comparability of scar outcomes across the cohort.

Although the sizes of the defects and flaps are reported in Table 2, the distribution of defect locations was heterogeneous, with certain anatomical regions (e.g., neck defects) represented by only a small number of cases. This imbalance may limit the ability to draw region-specific conclusions regarding outcomes and scar quality. In addition, differences in intrinsic skin properties—such as thickness, sebaceous density, vascularity, and mechanical tension—across various anatomical regions of the head and neck may influence wound-healing dynamics and scar formation. Because the present study included multiple anatomical sites without region-stratified analysis, direct comparison of healing outcomes between different facial and cervical regions may not be fully adequate.

In addition, detailed sociodemographic data, including patients’ occupation, place of residence, income level, and educational background, were not systematically collected in this retrospective dataset. These factors may influence patients’ perceptions of postoperative scarring and their acceptance of facial or scalp scars. Future prospective large-scale studies incorporating control groups and comprehensive sociodemographic variables are warranted to validate these findings and to better understand which patient populations are more likely to accept or be satisfied with visible postoperative scars.

It is also important to note that the classical and widely recognized applications of the KF include the reconstruction of lower extremity defects, particularly in the distal leg, foot, and flexor regions, where tissue laxity is limited and reconstructive challenges are substantial. However, these anatomical regions were beyond the scope of the present study. The primary objective of this study was not to compare hemi-KFs with other local flaps or to redefine the overall reconstructive hierarchy of keystone flaps, but rather to evaluate the clinical applicability and postoperative scar outcomes of hemi-keystone flap modifications specifically in head and neck defect coverage. Moreover, this study was not designed to establish definitive indications or an algorithm specifying when a KF should be preferred over other local flaps. Rather, flap selection was determined on a case-by-case basis according to defect characteristics, surrounding tissue laxity, and surgeon judgment. Future comparative or algorithm-based studies are required to more clearly define the optimal indications for hemi-KF application in head and neck reconstruction.

Furthermore, although this study proposes a structured categorization of hemi-KF techniques and their modifications, it was not intended to establish an entirely new classification system. Rather, the categorization presented herein represents a synthesis and practical reorganization of previously described hemi-KF concepts, based on our clinical experience, to facilitate clearer understanding and application in head and neck reconstruction. Future multicenter validation studies would be necessary to determine the broader applicability and reproducibility of this proposed framework.

Arguably, the inclusion of patients with a wide age range (9–92 years) is inappropriate because tissue repair capacity and wound-healing times differ significantly among children, adults, and older patients. While we acknowledge these physiological differences, we included all age groups to reflect real-world clinical practice. Age-stratified analysis was not performed in this study, but future studies with larger sample sizes should consider age-based subgroup analysis. A more detailed comparative analysis between adult and pediatric subgroups would be valuable to better elucidate age-related differences in wound healing and scar outcomes following hemi-KF reconstruction. Nonetheless, to the best of our knowledge, this study is significant, as it is the first case series to establish a classification system for the hemi-KF technique with its modifications. Furthermore, to the best of our knowledge, this is the first study to evaluate scars after hemi-KF reconstruction using the POSAS, and our findings may serve as a valuable reference for surgeons who intend to perform hemi-KF reconstruction, especially those concerned with postoperative scarring. Other factors such as cicatricial diathesis, nutritional status, and overall systemic health may influence healing outcomes; however, these were not specifically evaluated in this retrospective dataset. Thus, future studies should aim to include and assess these variables.

Finally, the mean flap surgery duration was 29.04 ± 14.56 min, with no case exceeding 2 h in this study. Postoperative complications are frequently correlated with prolonged operative durations and comorbid conditions such as diabetes, hypertension, obesity, advanced age, and renal disease [25,26,27]. A prior investigation documented heightened risks of cardiovascular, renal, and pulmonary complications with each additional hour of surgery beyond 6 h [26]. Furthermore, a previous study demonstrated that operative duration significantly impacts morbidity only beyond the 3 h mark, with the likelihood of complications escalating progressively after this critical threshold in plastic surgery [27]. This relatively short operative time supports the hemi-KF procedure as a valuable surgical technique that enhances safety by reducing the overall operative duration in patients with underlying medical conditions, such as diabetes mellitus and hypertension.

## 5. Conclusions

This study presents a simple, reliable, and versatile modification of the original hemi-KF, providing tailored reconstructive solutions for various defect sites in the head and neck region to achieve a more personalized approach to reconstructive surgery. In this retrospective case series of 50 patients, hemi-KF reconstruction demonstrated favorable scar outcomes, low complication rates, and acceptable operative times, supporting its clinical feasibility for small to moderate-sized head and neck defects. The technique may be particularly useful in cases where adjacent tissue laxity allows local advancement while preserving vascular reliability, offering the advantages of technical simplicity, reproducibility, and satisfactory aesthetic outcomes. However, its application requires careful case selection, and limitations include operator dependency, anatomical heterogeneity, and variability in follow-up duration, as discussed above. Our experience extends the application of hemi-KF reconstruction to plastic surgery. Rather than establishing a new classification system, this study provides a structured clinical framework that may assist surgeons in selecting and applying hemi-KF modifications more systematically. Future prospective, multicenter, and comparative studies are necessary to further define optimal indications, advantages, and potential limitations of hemi-KF reconstruction. In addition, we plan to expand the scope of application to include the reconstruction of defects in body regions beyond the head and neck in future research.

## Figures and Tables

**Table 1:** Demographic and anamnestic data of patients.

Characteristics	Value
Total cases, *n*	50
Male sex, *n* (%)	36
Female sex, *n* (%)	14
Age, years, mean ± SD	49.16 ± 22.02
Comorbidities (history of underlying diseases), *n* (%)	30 (60%)
Diabetes	10 (20%)
Hypertension	21 (42%)
Anticoagulants (aspirin)	13 (26%)
Smoking	15 (30%)

SD, standard deviation.

**Table 2:** Summary of characteristics and outcomes following reconstruction with the hemi-keystone flap and its modifications.

Characteristic	Value
Total flaps	50
Defect cause	
Trauma	33
Skin tumor	10
Postoperative wound dehiscence	3
Skin tumor	2
Skin infection	2
Defect location	
Scalp and forehead	16
Nose	11
Cheek	5
Eyelid area	5
Lip area	4
Auricular area	7
Neck area	2
Defect size (cm^2^), mean ± SD	4.68 ± 4.14
Flap size (cm^2^), mean ± SD	11.79 ± 16.69
Flap type	
Single original hemi-KF	30 (60%)
Single rounded hemi-KF	4 (8%)
Type A double hemi-KF	2 (4%)
Type B double hemi-KF	11 (22%)
Type C double hemi-KF	3 (6%)
Flap surgery time (min), mean ± SD	29.04 ± 14.56
Postoperative complications	
Flap failure	0
Infection	0
Hematoma/seroma	0
Dehiscence	3 (6%)
Follow-up period, months, mean ± SD	6.34 ± 5.43
PSAS total score, mean ± SD	15.30 ± 3.59
OSAS total score, mean ± SD	17.12 ± 3.70
Overall patient satisfaction, mean ± SD	3.38 ± 0.87
Objective scar rating, mean ± SD	3.98 ± 1.02

SD, standard deviation; KF, keystone flap; OSAS, Objective Scar Assessment Scale; PSAS, Patient Scar Assessment Scale.

**Table 3:** Comparison of the PSAS total scores, OSAS total scores, overall patient satisfaction, and objective scar rating between male and female patients.

	Mean PSAS Total Score	Mean OSAS Total Score	Mean OPS Score	Mean OSR Score
Male patients	15.36 ± 3.60	17.22 ± 3.67	3.40 ± 0.86	4.02 ± 0.98
Female patients	15.08 ± 3.60	17.28 ± 3.79	3.34 ± 0.89	4.02 ± 0.10
*p*-value	0.08	0.13	0.74	0.08

OSAS, Objective Scar Assessment Scale; PSAS, Patient Scar Assessment Scale; OPS, overall patient satisfaction; OSR, objective scar rating. All variables are expressed as means ± standard deviations. The significance level was set at a *p*-value of <0.05.

**Table 4:** Comparison of the PSAS total scores, OSAS total scores, overall patient satisfaction, and objective scar rating between patients who underwent single hemi-KF reconstruction and those who underwent double hemi-KF reconstruction.

	Mean PSAS Total Score	Mean OSAS Total Score	Mean OPS Score	Mean OSR Score
Single hemi-KF	15.29 ± 3.60	17.16 ± 3.77	3.37 ± 0.89	3.97 ± 1.04
Double hemi-KF	15.38 ± 3.67	17.10 ± 3.64	3.42 ± 0.87	4.00 ± 1.00
*p*-value	0.21	0.33	0.08	0.55

KF, keystone flap; OSAS, Objective Scar Assessment Scale; PSAS, Patient Scar Assessment Scale; OPS, overall patient satisfaction; OSR, objective scar rating. All variables are expressed as means ± standard deviations. The significance level was set at a *p*-value of <0.05.

## Data Availability

The original contributions presented in this study are included in the article. Further inquiries can be directed to the corresponding author.

## Abbreviations

The following abbreviations are used in this manuscript:

KF	Keystone flap
OVC	Omega variation closure
SMUM	Sydney Melanoma Unit modification
POSAS	Patient and Observer Scar Assessment Scale
PSAS	Patient Scar Assessment Scale
OSAS	Observer Scar Assessment Scale
OSR	Objective scar rating
OPS	Overall patient satisfaction
SD	Standard deviation
